# A Re-Audit of the Assessment of the Nutritional Status of Patients Admitted to the General Adult Inpatient Wards in Mersey Care NHS Foundation Trust

**DOI:** 10.1192/bjo.2024.576

**Published:** 2024-08-01

**Authors:** Declan Hyland, Faraaz Abulais, Ranjan Baruah

**Affiliations:** Mersey Care NHS Foundation Trust, Liverpool, United Kingdom

## Abstract

**Aims:**

Obesity and malnutrition have increased prevalence in individuals with mental disorder. Patients with severe mental illness are at increased likelihood of developing weight-related comorbidities, particularly type II diabetes mellitus.

Admission to the psychiatric ward provides an opportunity to address, not only the patient's mental health issues, but also any physical health issues.

The aim of this re-audit was to assess whether patients were managed in compliance with the Mersey Care NHS Foundation Trust Nutritional Screening Protocol on eight general adult inpatient wards across the Trust.

**Methods:**

Data from the first five admissions (starting from 1^st^ April 2023) to eight general adult inpatient wards in the Trust was collected and assessed.

A total of 40 inpatient admissions were identified. The results were collated and compared to the standard – Mersey Care's Nutritional and Hydration Policy.

**Results:**

36 patients (90%) had a Malnutrition Universal Scoring Tool (MUST) completed within 72 hours of admission. Of the four patients (10%) who didn't have a MUST score within 72 hours of admission, three were completed after 72 hours.

46% of patients had a MUST score of 0 (low risk), 31% a MUST score of 0 (high risk obesity), 10% a MUST score 1 (medium risk) and 13% a MUST score of 2 or above (high risk).

Of the five patients with a MUST score of 2 or above (high risk), three (60%) were compliant with all elements of the Nutrition Screening Tool Care Plan. Of the 12 patients with a MUST score of 0 (high risk obesity), seven (58%) were compliant with all elements. Of the four patients with a MUST score of 1 (medium risk), all were compliant with all elements.

Overall, 31 (79%) patients had every element of the Nutrition Screening Tool Care Plan completed.

**Conclusion:**

There was significant assurance of systems and processes in place and working well to ensure compliance, with only minor issues of concern identified.

Whilst the MUST score within the first 72 hours following admission had been completed in most inpatients, referrals to the dietician had not been done consistently in line with Trust policy. This is an area that requires addressing. Some training may need to be delivered to underline the importance of adhering to Trust policies.

An action plan to circulate the audit findings to all general adult inpatient wards across the Trust and re-auditing with a larger sample size across the Trust has been recommended.

